# Stable Cellulose Nanofibril Microcapsules from Pickering Emulsion Templates

**DOI:** 10.1021/acs.langmuir.1c03025

**Published:** 2022-03-09

**Authors:** Hui Shi, Kazi M. Zakir Hossain, Davide Califano, Ciaran Callaghan, Ekanem E. Ekanem, Janet L. Scott, Davide Mattia, Karen J. Edler

**Affiliations:** † Department of Chemistry, University of Bath, Claverton Down, Bath BA2 7AY, U.K.; ‡ Centre for Sustainable Chemical Technologies, University of Bath, Claverton Down, Bath BA2 7AY, U.K.; § Department of Chemical Engineering, University of Bath, Claverton Down, Bath BA2 7AY, U.K.

## Abstract

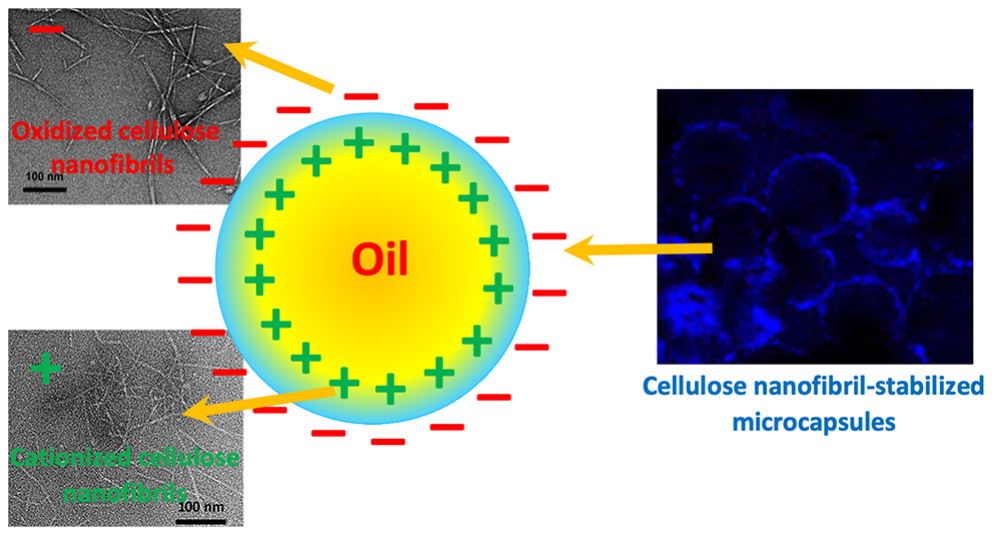

Electrostatic attractions are essential in any complex formation between the nanofibrils of the opposite charge for a specific application, such as microcapsule production. Here, we used cationized cellulose nanofibril (CCNF)-stabilized Pickering emulsions (PEs) as templates, and the electrostatic interactions were induced by adding oxidized cellulose nanofibrils (OCNFs) at the oil–water interface to form microcapsules (MCs). The oppositely charged cellulose nanofibrils enhanced the solidity of interfaces, allowing the encapsulation of Nile red (NR) in sunflower oil droplets. Microcapsules exhibited a low and controlled release of NR at room temperature. Furthermore, membrane emulsification was employed to scale up the preparation of microcapsules with sunflower oil (SFO) encapsulated by CCNF/OCNF complex networks.

## Introduction

Microcapsules (MCs) have an extensive range of applications as carriers and release vehicles for drugs, cosmetics, dyes, and many other bioactive/functional ingredients.^1,2^ However, the vast majority of microcapsules are manufactured using nonbiodegradable polymers obtained from nonrenewable sources.^3,4^ With growing awareness of pollution arising from microplastics and fossil fuels, polysaccharide-based microcapsules have attracted considerable attention as these are biodegradable, renewable, and biocompatible.^5,6^ Compared with synthetic biopolymers or heavily modified biomolecules, the utilization of polysaccharides can reduce the burden on the environment, lower the cost, and increase the industrial use of these microcapsules.^7,8^ However, the production of polysaccharide-based microcapsules is not a straightforward process. Many reported methods employ complex preparation methods, such as multiple deposition steps, cross-linking of biopolymers,^9,10^ or decomposition of template cores after coating biopolymers.^11,12^ Furthermore, most of the microcapsule production techniques reported in the literature are of low feasibility for scale-up industrial production.^13^ In contrast, membrane emulsification (ME) is a well-controlled and highly engineered process that satisfies the essential requirements for scaling-up manufacturing volumes.^14,15^ With further postprocessing, emulsions prepared via membrane emulsification can be turned into microcapsules for the delivery of actives.^16,17^ Furthermore, a much lower shear force is generated in ME compared to other emulsification processes, which allows the encapsulation of sensitive materials without damage.^18^

Recently, microcapsules prepared by electrostatic interaction between polysaccharides have drawn more attention.^19−21^ Among all of the polysaccharides used, cellulose is the most abundant organic polymer on earth.^22^ It is obtained largely from plants where components of the biomass can be removed, leaving cellulose structures that can be further broken down mechanically or chemically to create particles that are nanometers in diameter but up to microns long depending on the preparation method. The high density of surface hydroxyl groups on the cellulose surface enables various chemical functionalization reactions such as oxidation^23,24^ and cationization^25^ to tweak their surface charge profiles.^26^ Incorporating a specific amount of charge density on the cellulose surface is crucial to provide the necessary electrostatic repulsion forces to enable the proper dispersion of cellulose fibers in an aqueous medium.^27^

In this work, the electrostatic attraction of oppositely charged cellulose nanofibrils (CCNFs) was used in situ at the oil–water interface of an oil-in-water (O/W) emulsion. The formation of cellulose nanofibril complexes at the oil–water interface was found to stabilize emulsion droplets. The effect of introducing oppositely charged cellulose nanofibrils to the Pickering emulsion (PE) template was investigated via dye release studies under static (diffusion) and force field conditions (centrifugation and mechanical stirring) at room temperature. The dye release percentage from the most stable microcapsules was also investigated after conditioning at various pH environments (4.0, 5.0, 6.5, and 8.5). Membrane emulsification (ME) was applied to prepare microcapsules using bioderived cellulose particles and natural sunflower oil (SFO), proving the feasibility of scaling-up production of sustainable and biodegradable microcapsules.

## Experimental Section

### Materials

α-Cellulose powder (C8002), glycidyl trimethylammonium chloride (GTMAC, ≥90%), 0.1 M AgNO_3_ aqueous solution (≥95%), calcofluor white stain (18909), Nile red (NR), sodium hydroxide pellets (≥98%), and hydrochloric acid (EMPLURA 32%) were purchased from Merck, U.K. and used as received. Pure sunflower oil made from 100% sunflower seeds was purchased from TESCO UK (pack size: 3 L). Ultrapure DI water (18.2 MΩ cm) was used for all dilutions and sample preparation. Oxidized cellulose nanofibrils (OCNFs) as a ca. 8 wt % solids paste in water with a degree of oxidation degree of 25% were prepared via 2,2,6,6-tetramethylpiperidinyloxy (TEMPO)-mediated oxidation as previously reported.^28,29^

### Experiments

#### Preparation and Characterization of Modified Cellulose Nanofibrils

CCNF was produced by modifying α-cellulose with GTMAC following the semidry procedure using 3 mol equivalents of GTMAC relative to cellulose anhydroglucose units, as previously reported.^25,28^ For the purification process after cationization, the paste was first washed with absolute ethanol (50%) followed by DI water and centrifugation at 8000 rpm for 5 min (three cycles for each solvent). After each cycle, after discarding the supernatant, the sediment was redispersed in fresh solvent for repeated washing. The prewashed CCNF dispersion was dialyzed against DI water for 5 days (medium replaced twice daily). The purified CCNF dispersion was freeze-dried, and the degree of substitution (DS) of cationic cellulose was determined to be 20% by conductometric titration of chloride ions (trimethylammonium chloride groups) with AgNO_3_ aqueous solution (see Figure S1).^25,28^

The OCNF was further purified via dialysis under ultrapure DI water to remove any salt and preservatives, as described elsewhere.^30^ Briefly, approximately 20 g of OCNF slurry was dispersed in 100 mL of deionized water and stirred at room temperature for 30 min. After being acidified to pH 3 using 1 M aqueous HCl solution, the OCNF dispersion was dialyzed against DI water (cellulose dialysis tubing molecular weight cut-off (MWCO) 12,400) for 5 days (medium replaced twice daily). The dialyzed OCNF dispersion was homogenized at 6500 rpm for 30 min using an Ultra Turrax (IKA T25 digital) and adjusted to pH 7 using 0.1 M NaOH. After a second dialysis step for an additional 3 days (medium replaced twice daily), the purified OCNF dispersion was freeze-dried.

#### Cellulose Dispersion Preparation

The CCNF dispersions (0.05 and 0.1 wt %) were prepared by dispersing the required amount of freeze-dried CCNF in DI water using a sonication probe (Ultrasonic Processor, FB-505, power 500 W, using 1 s on/off pulses for a net time of 10 min at an amplitude of 30% in an ice bath for 20 mL of the dispersion). OCNF dispersions of varying concentrations (0.05, 0.2, and 0.5 wt %) were prepared using the same sonication protocol used for CCNF dispersions.

The morphology of the OCNF and CCNF were characterized using transmission electron microscopy (TEM, JEOL, JEM-2100 Plus) at an operating voltage of 200 kV. A dilute OCNF or CCNF suspension (0.01 wt %) was added onto a Cu grid (mesh size 300) and then negatively stained using uranyl acetate (from Merck, U.K.) (2 wt %) for enhanced contrast during TEM measurements.

#### Preparation of Microcapsules via the Pickering Emulsion Template

**Figure 1:**
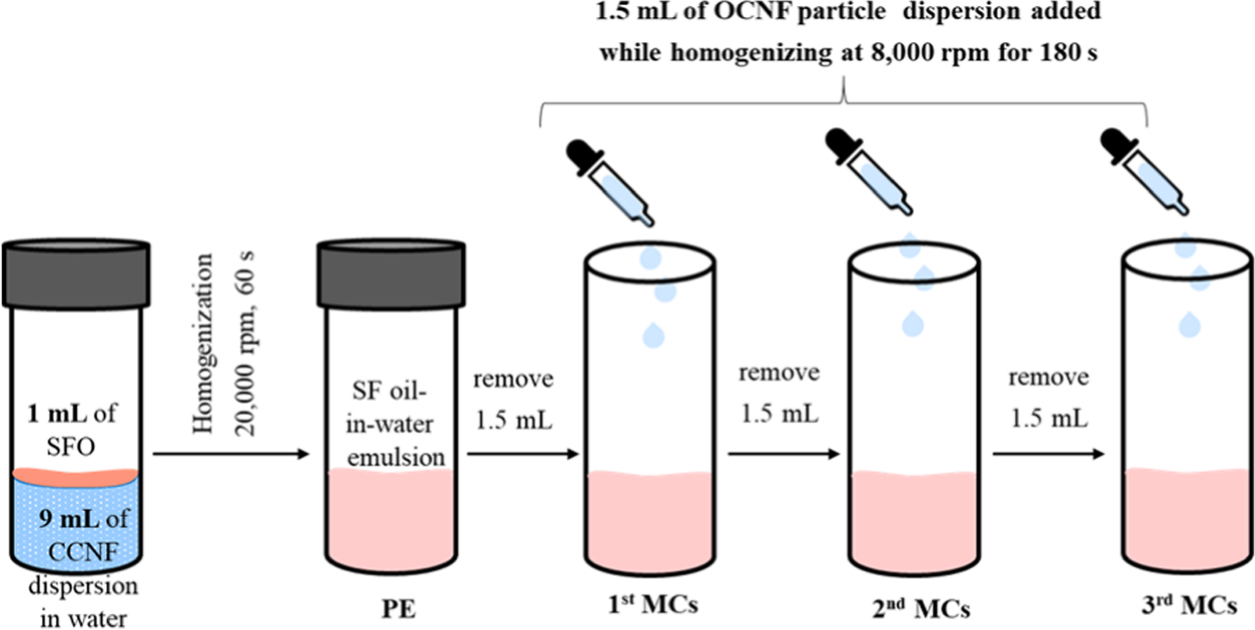
Schematic of primary Pickering emulsion (PE) and microcapsule (MC) preparation protocol.

This study prepared microcapsules by encapsulating sunflower oil (SFO) using oppositely charged cellulose nanoparticles. SFO is a plant-sourced oil as well as edible, hence chosen in this study along with bioderived functionalized cellulose nanoparticles for sustainable microcapsules preparation. First, CCNF-stabilized Pickering emulsions (PEs) were prepared by blending 1 mL of sunflower oil (SFO) and CCNF dispersion (9 mL of 0.05 or 0.1 wt % dispersion) using an Ultra Turrax (IKA T25 digital) at 20 000 rpm for 1 min at room temperature (Figure 1). The first microcapsules (first MC) were prepared by replacing 1.5 mL of CCNF-stabilized PE with 1.5 mL of oppositely charged OCNF dispersion (0.05, 0.2, or 0.5 wt % dispersion added dropwise) while homogenizing at 8000 rpm for 3 min at room temperature. Then, the second and third MCs were subsequently prepared by successive replacement of 1.5 mL of previous microcapsules with an equal amount of OCNF dispersion (Figure 1). Typically, 1.5 mL of PE or MCs was replaced with the same amount of fresh OCNF dispersion at every successive stage of MCs preparation, to retain the final volume of the MC sample at 10 mL to maintain similar shear forces during the homogenization process.

### Characterization

#### Microscopic Imaging

Optical images of the PE and MCs were taken after homogenization using an EVOS AMG (AMF 4300) microscope, and their stability was monitored visually over time (for any creaming/phase separation). The stability of the PE and MCs was further confirmed via confocal imaging (ZEISS; LSM50META), where the oil phase was dyed with NR and cellulose fibrils were stained with calcofluor white.

#### Surface Charge

The ζ-potential of the OCNF (0.05 wt %), CCNF (0.05 wt %), PE, and MC suspensions was measured using a Zetasizer (Malvern Zetasizer Nano ZSP, U.K.). PE/MC samples (100 μL) were diluted using DI water (900 μL) before the measurement. The samples (∼850 μL) were injected in the folded capillary electrode cell and equilibrated at 25 °C for 120 s before measuring as an average of 5 from 100 scans each. The ζ-potential was calculated using Henry’s law utilizing the Smoluchowski model loaded in the Zetasizer software (Malvern).

#### Quantification of Nile Red (NR) Release

For the dye release study, NR was dissolved in SFO at a weight fraction of 0.00275 wt %. The Pickering emulsions and various microcapsules (first, second, and third MCs) were prepared in a 50 mL centrifuge tube (Falcon) according to the method illustrated in Figure 1. Then, 5 mL of pure sunflower oil (NR-free) was added carefully on the top of the PE and MCs (10 mL) suspension along the tube wall. These were then stored at room temperature, allowing for any NR to diffuse from the PE or MC phase to the top oil layer. The absorbance of the upper oil layer was measured using a UV–visible spectrophotometer (Agilent 8453) at 520 nm after 1 and 7 days. The NR released was calculated from the calibration curve (Figure S2), and the percentage of dye release with respect to its initial concentration was reported.

Dye release studies were also conducted under different applied force fields, namely, centrifugation and mechanical stirring at room temperature. In the case of centrifugation, the abovementioned PE and MCs (10 mL of sample + 5 mL of dye-free fresh oil) were centrifuged at 8000 rpm for 10 min and then allowed to diffuse for 1 and 7 days before taking the absorbance of the upper oil layer at 520 nm. For mechanical stirring studies, the samples were stirred at 2000 rpm for 10 min using an overhead stirrer (propeller dimension 14.5 mm × 12 mm and Falcon tube internal diameter ∼27 mm) and then allowed to diffuse for 1 and 7 days. To separate the oil layer from the blend, a short centrifugation (8000 rpm for 2 min) was done after 1 and 7 days just before the absorbance measurement. The protocols used for all dye release studies (diffusion, centrifugation, and mechanical stirring) are shown in Figure S3.

In addition, a dye release study on the most stable MCs (third MCs) was done in different pH environments (4.0, 5.0, 6.5, and 8.5). The pH of the as-prepared MCs was ∼6.5 (without any adjustment); therefore, the lower and higher pH values were obtained using HCl (0.1 M) and NaOH (0.1 M) solution, respectively.

All dye release experiments were repeated in triplicate, and error bars report the standard deviation for each data point.

#### Membrane Emulsification

A micropore LDC-1 dispersion cell (Micropore Technologies Ltd.), a 72–2685 digital-control DC power supply (Tenma 72-2685 Digital-Control power supply, 30 V, 3 A), a membrane with 30 μm pores (Micropore Technologies Ltd.), and a syringe pump were used for the membrane emulsification process. Around 45 mL of 0.1 wt % CCNF dispersion was used as the continuous phase, and stirring was applied by adjusting the power supply to 6 V (∼530 rpm). Then, 3.75 mL of sunflower oil stained with NR was injected at a speed of 0.1 mL/min from a 20 mL syringe through the membrane as the dispersed phase. The produced oil droplets were stabilized by CCNF in the continuous phase forming the primary Pickering emulsion (PE). After injecting all of the dispersed phase, 20 mL of 0.5 wt % oppositely charged OCNF aqueous dispersion was injected at 0.1 mL/min into the CCNF-stabilized primary emulsion while stirring at 6 V (∼530 rpm) to prepare the microcapsules. The microcapsules were then collected, and optical images were taken just after preparation and after a week of storage.

## Results and Discussion

### Morphology of OCNF and CCNF

**Figure 2:**
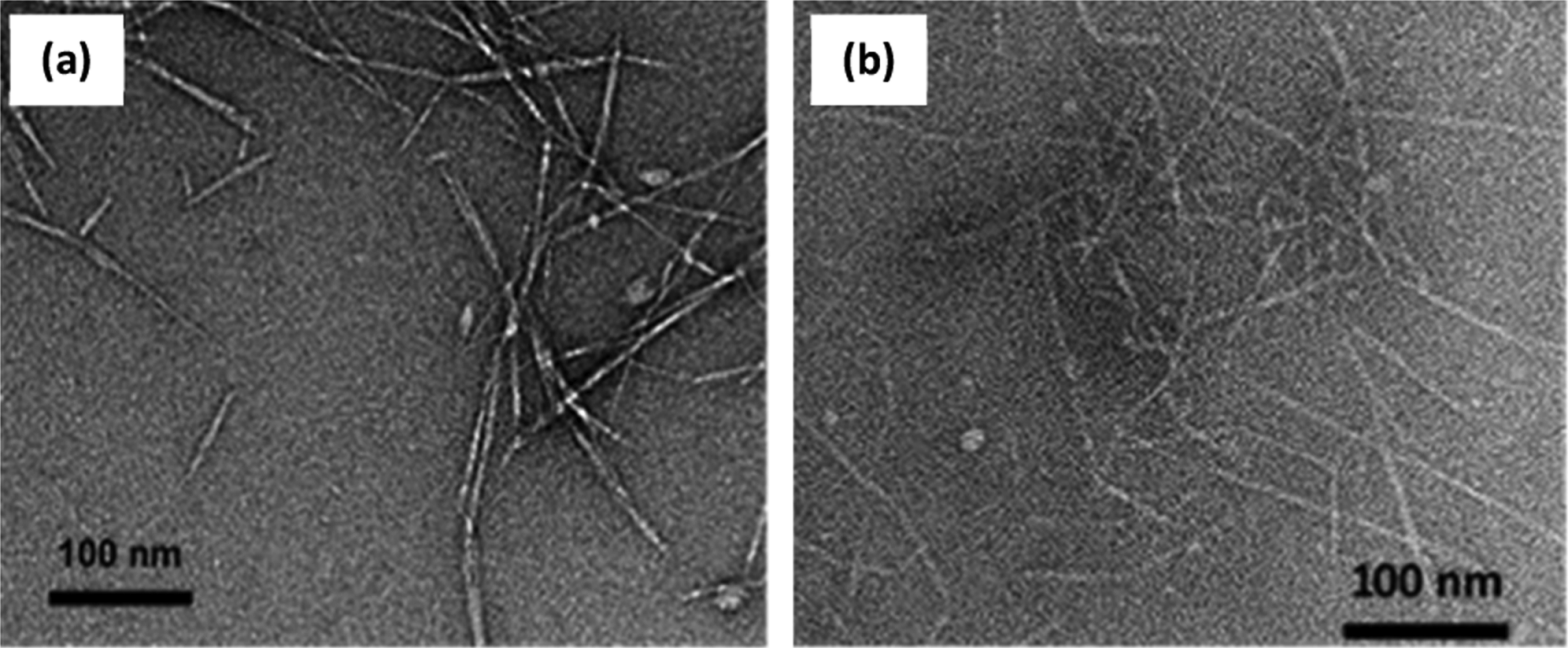
TEM micrographs of (a) OCNF and (b) CCNF dispersion in DI water at 0.01 wt %. Uranyl acetate (2%) was used for contrast imaging.

TEM micrographs, presented in Figure 2a,b, revealed fibrillar particles of OCNF and CCNF. Both materials have been previously characterized with a length (*L*) of 160 ± 60 nm and a diameter (*D*) of 7 ± 2 nm for OCNF (statistical image analysis of TEM micrographs from averaging 175 measurements)^29^ and *L* = 105 ± 35 nm and *D* = 7 ± 2 nm for CCNF (measurements of over 100 particles).^31^

### Interactions of CCNF-Stabilized Pickering Emulsion with OCNF at the Oil–Water Interface

**Figure 3:**
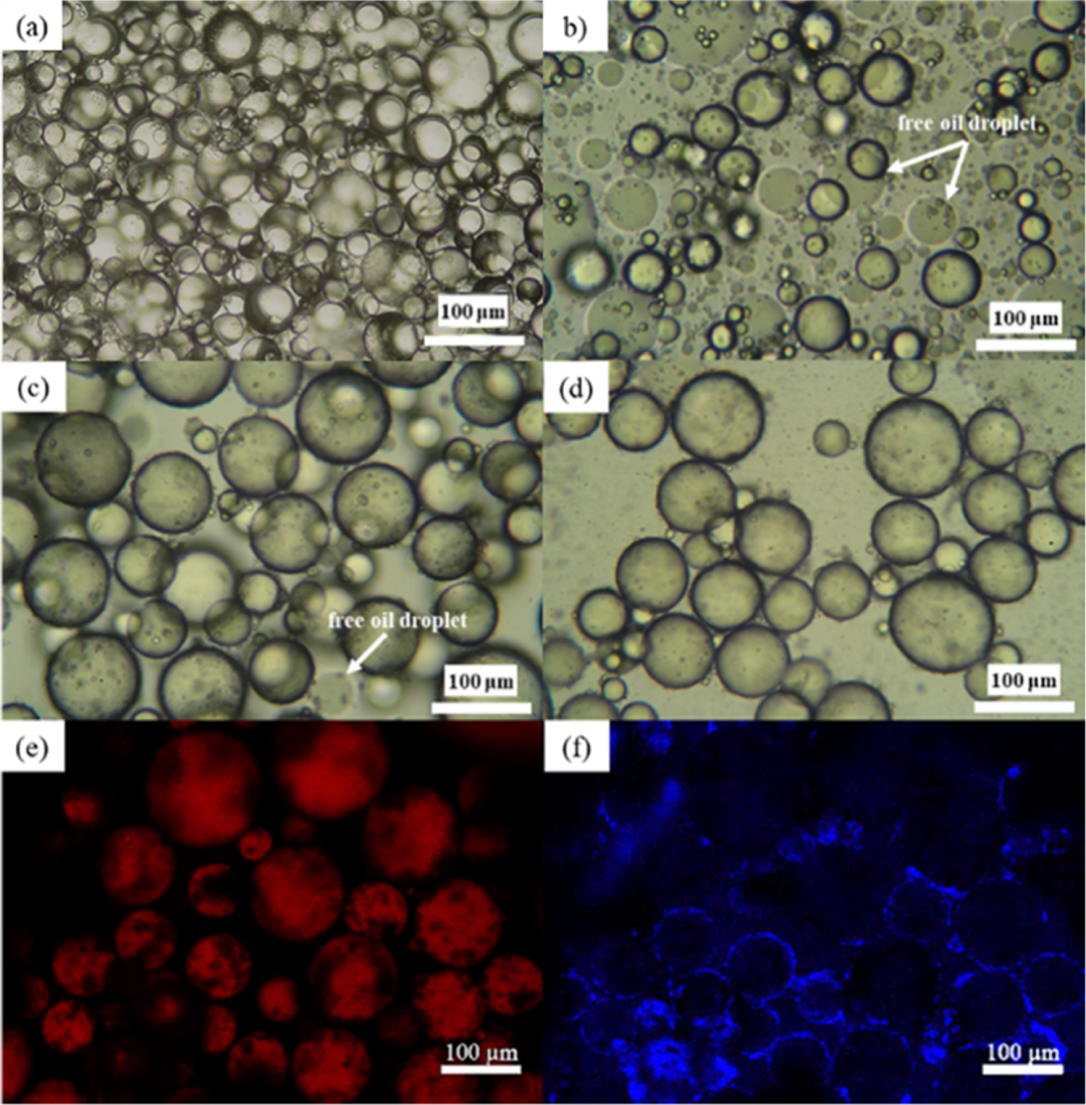
Optical microscope images of the PE and MCs prepared by replacing 1.5 mL of CCNF (0.05 wt %)-stabilized PE with an equal amount of 0.05 wt % OCNF dispersion: (a) control CCNF (0.05 wt %)-stabilized PE before adding any OCNF dispersion, (b) first MCs, (c) second MCs, and (d) third MCs. Images were taken just after preparation and (e, f) confocal microscope images of third MCs after standing at room temperature for 1 week (sunflower oil dyed with NR showing in red and cellulose stained with calcofluor white stain showing in blue).

The primary oil-in-water (O/W) emulsion stabilized with CCNF was used as a template for the adsorption of the second layer of OCNF. A CCNF dispersion of 0.05 wt % was used to generate O/W primary PE, which remained stable after 1 week of storage at room temperature. This is attributed to the fact that hydrophobic CCNF (contact angle of water on the glass slide coated with CCNF = 95 ± 3°, see Figure S4) is stable at the O/W interface. An OCNF dispersion of 0.05 wt % was added stepwise to the CCNF-stabilized PE following the protocol in Figure 1. Results show that there are free oil droplets in the first MC sample after the first addition of OCNF compared to the PE (Figure 3a,b). This suggests that the stabilizing ability of CCNF/OCNF complexes at the oil–water interface is lower than that of the CCNF alone. Upon adding more OCNF, droplet coalescence was hindered, producing fewer free oil droplets (second MCs in Figure 3c). In the third MC sample, droplets were stable with no oil released (Figure 3d). As seen from the confocal images of the third MC sample (Figure 3e,f), most cellulose gathers at the surface of the droplets, preventing coalescence of the oil droplets; they retain their spherical shapes, showing the significance of the CCNF/OCNF interaction.

The first and second MCs were still positively charged, suggesting an insufficient amount of the OCNF interacted with the CCNF at the oil–water interface (Table 1). Hence, coalescence was observed in the optical micrographs for these samples. However, in the case of the third MCs, charge inversion was observed (Table 1), and droplets were found to be stable as observed via the confocal laser microscopic image (Figure 3e,f). The attributions “stable” and “unstable” in Table 1 are based on the visual observation of the MC samples after storage at room temperature for 1 week: unstable (visible free oil phase) or stable (no free oil phase), as shown in Figure S5.

**Table 1:** Summary of the Interaction between CCNF (0.05 wt %)-Stabilized Emulsion with OCNF

CCNF stock (wt %)	OCNF stock (wt %)	samples (MCs were prepared according to the scheme presented in Figure 1 using CCNF and OCNF stock dispersions)	ζ-potential (mV)	CCNF/OCNF mass ratio in MCs (see Table S1)	phenomenon
0.05	-	CCNF dispersion	+42 (±2)		stable
-	0.05	OCNF dispersion	–60 (±4)		stable
0.05	-	PE	+38 (±2)		stable
	0.05	first MCs	+35 (±1)	5.10	unstable
		second MCs	+21 (±2)	2.34	unstable
		third MCs	–42 (±2)	1.43	stable

**Figure 4:**
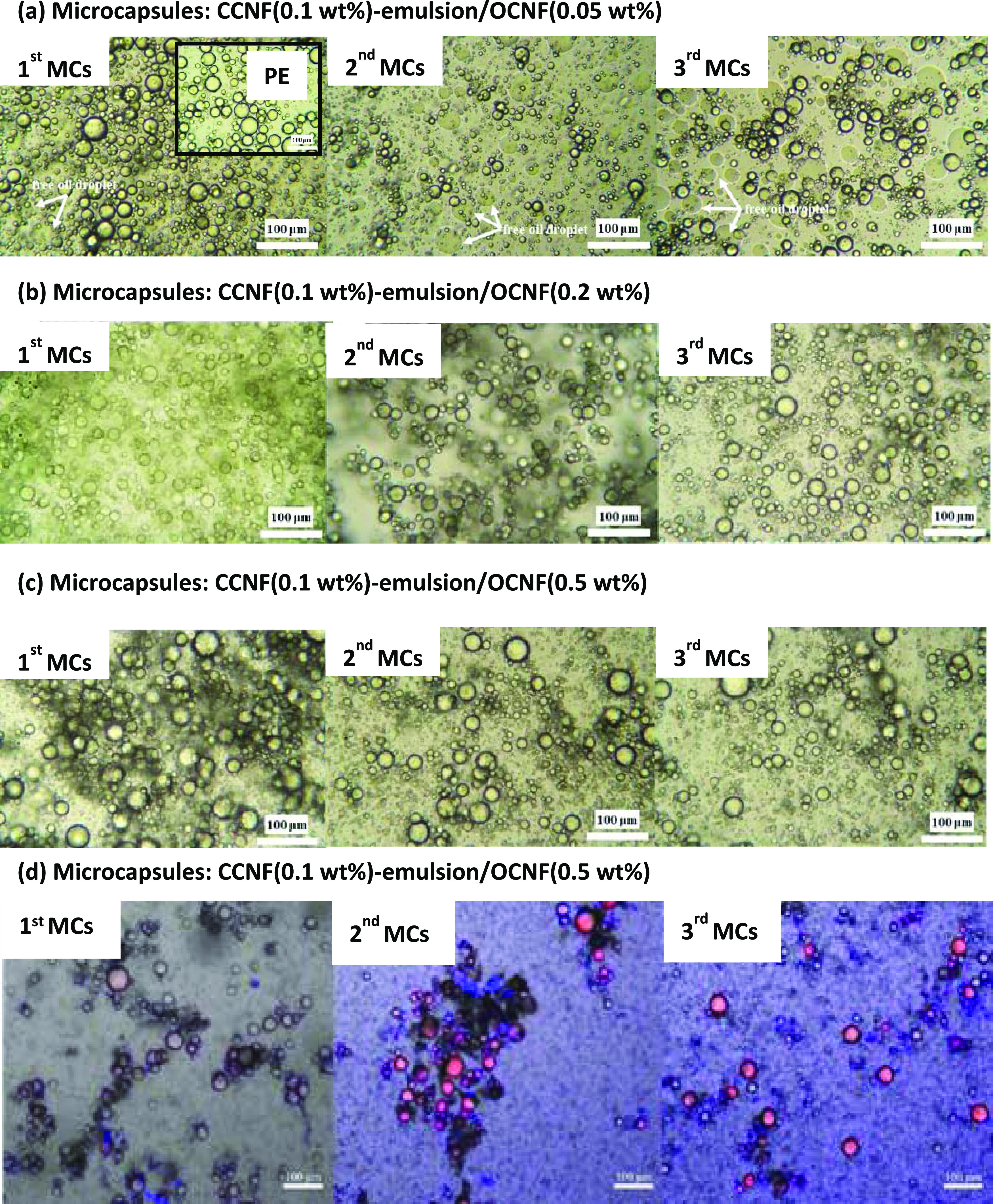
Optical microscope images of microcapsule samples prepared using CCNF (0.1 wt %)-stabilized emulsion as a template with the various concentrations of OCNF dispersion: (a) MCs prepared using 0.05 wt % OCNF dispersion (inset shows the control CCNF (0.1 wt %)-stabilized PE). (b) MCs prepared using 0.2 wt % OCNF dispersion. (c) MCs prepared using 0.5 wt % OCNF dispersion; optical images were taken just after preparation. (d) Confocal microscope images of MC samples (c) after standing at room temperature for 1 week (SFO dyed with NR showing red and cellulose stained with calcofluor white stain showing blue).

A higher concentration of CCNF (0.1 wt %) dispersion was also utilized with SFO to prepare another primary PE. At this concentration, the CCNF (0.1 wt %)-stabilized primary emulsion was more stable against significant coalescence (Figure 4a). OCNF dispersions of 0.05 and 0.5 wt % were used to interact with the CCNF (0.1 wt %)-stabilized emulsions to form microcapsules, according to the protocol in Figure 1. The interaction of OCNF at lower concentration (0.05 wt %) with the primary PE at different stages (first, second, third MC samples) resulted in the coalescence of droplets and release of free oil, as observed via microscopic images in Figure 4b. This reveals that the transformation of CCNF to OCNF/CCNF complexes at the interface negatively influences emulsion stability. Moreover, the ζ-potential decreased from +37 to +12 mV (Table 2). Although an insufficient amount of OCNF was added to cause a reversal of the surface charge of droplets, the decrease of ζ-potential with increasing OCNF confirms the interfacial electrostatic interaction.

**Table 2:** Summary of the Interaction between the Various Concentrations of OCNF with CCNF (0.1 wt %)-Stabilized PE

CCNF stock (wt %)	OCNF stock (wt %)	samples (MCs were prepared according to the scheme presented in Figure 1 using CCNF and OCNF stock dispersions)	ζ-potential (mV)	CCNF/OCNF mass ratio in MCs (see Table S1)	phenomenon
0.1	−	PE	+40 (±1)		stable
	0.05	first MCs	+37 (±2)	10.20	unstable
		second MCs	+33 (±3)	4.68	unstable
		third MCs	+12 (±1)	2.87	unstable
0.1	0.2	first MCs	+31 (±2)	2.55	unstable
		second MCs	–22 (±1)	1.17	stable
		third MCs	–45 (± 4)	0.71	stable
0.1	0.5	first MCs	–45 (±2)	1.02	stable
		second MCs	–56 (±2)	0.47	stable
		third MCs	–60 (±4)	0.29	stable

To prevent coalescence, the concentration of the added OCNF was increased to 0.2 wt %. The ζ-potential values of the first and third MC samples in this set were reversed from +31 to −45 mV (see Table 2), reflecting the adsorption of OCNF particles at the CCNF interface. Using an even higher OCNF concentration (0.5 wt %), the ζ-potential values of all microcapsules (first, second, third MC samples) reversed from positively charged to negatively charged with stability unchanged (Table 2). The second and third MC samples achieved ζ-potential values similar to the pure OCNF in DI water (−60 mV), which might be due to the complete coverage of CCNF-stabilized oil droplets and the surrounding particles by the oppositely charged OCNF. Thus, the surface charge of the droplets can be reversed in a more controlled way by adjusting the concentrations of CCNF and OCNF. Once coalescence is prevented, a further dilution or washing process does not affect the stability, as confirmed via confocal imaging (Figure 4d). This implies that the adsorption of OCNF/CCNF complexes at the interface is irreversible. Further addition of oppositely charged cellulose particles with respect to the particle’s charge of the outer surface, for example by adding another layer of CCNF, would increase the shell thickness, potentially forming stronger MCs. However, this would include additional preparation steps, making the process more complex.

MCs prepared using the same materials but added in the opposite order, an OCNF-stabilized PE template followed by adsorption of CCNF, revealed good dispersion for the control OCNF-stabilized PE (Figure S6); however, after 1 week of storage at room temperature, the primary OCNF-stabilized PE and all of the MCs showed clear phase separation. This destabilization may be due to the high surface charge of OCNF (−60 ± 4 mV). Since the repulsion between OCNF is strong, they are easily removed from the interface, and hence a low surface coverage induces coalescence of the oil droplets. Furthermore, cellulose aggregates were observed in the water phase (freed from the oil–water interface), as shown in the confocal image (Figure S6). This might be due to the hydrophilic nature of the OCNF (contact angle of water on the glass slide coated with OCNF = 41 ± 2°, see Figure S4), which are poorly attached to the oil–water interface of the emulsion.

### Dye Release from Microcapsules

**Figure 5:**
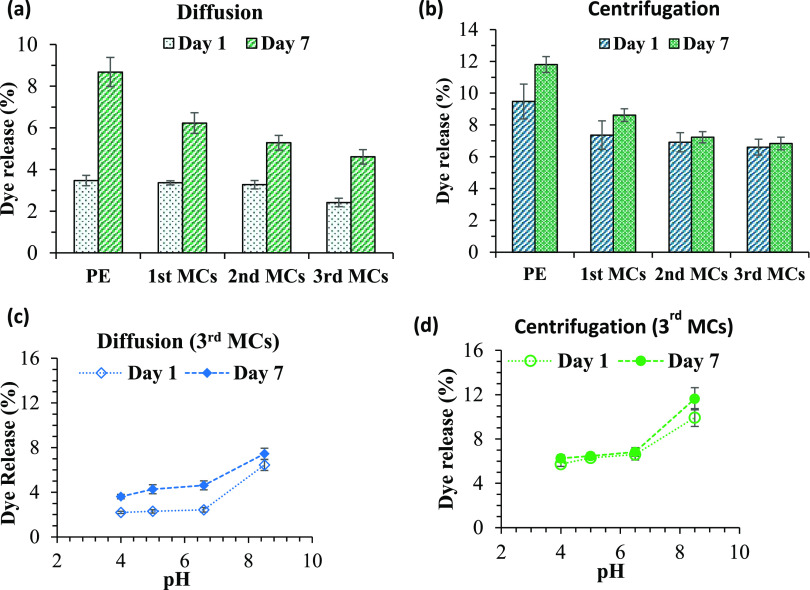
Variation of the NR release from the primary CCNF (0.1 wt %)-stabilized PE and the MCs prepared using 0.5 wt % OCNF dispersion: (a) via diffusion and (b) via centrifugation (8000 rpm for 10 min). Dye release percentage of the third MCs in various pH environments: (c) via diffusion and (d) via centrifugation (8000 rpm for 10 min).

Here, the most stable formulation, i.e., the CCNF (0.1 wt %)-stabilized PE and the associated MCs prepared using 0.5 wt % OCNF dispersion (see Table 2), was used for dye release studies using the lipophilic stain NR as an active substance in the oil phase. The release of NR from these microcapsules can be attributed to two distinct mechanisms: first, the release could occur during coalescence of the droplets due to poor encapsulation caused by weak CCNF/OCNF interactions at the oil–water interface. Second, in droplets with stable and solid encapsulation (CCNF (0.1 wt %)-stabilized emulsion/OCNF complex), the diffusion of dye from the capsule core through the cellulose shell could also induce dye release. Figure 5a shows the dye release percentage variation via diffusion for the CCNF (0.1 wt %)-stabilized PE and the stable first, second, and third MCs kept at room temperature for 7 days. After 1 day, the PE released ∼3.5 wt % dye and the first and second MCs did not show any significant reduction in dye release compared to the PE. However, the third MCs showed a noticeable decrease in dye release percentage (∼2.4%) compared to the PE and first and second MCs. After 7 days of storage at room temperature, the primary PE released ∼8.7% dye, whereas the first, second, and third MCs released ∼6.2, 5.2, and 4.6% dye, respectively. This suggests that with the increasing concentration of OCNF, the interaction of OCNF/CCNF at the CCNF-stabilized O/W interface increased, leading to better encapsulation.

As the third MC samples were found to be more stable, only these MCs were considered in further studies under varying pH environments. With decreasing pH, after 1 day of storage at room temperature, the dye release percentage of the third MCs decreased marginally, from ∼2.4% (at pH 6.5) to ∼2.2% (at pH 4.0); however, after 7 days, the dye release percentage reduced from ∼4.6% (at pH 6.5) to ∼3.6% (at pH 4.0), as can be seen in Figure 5c. A further solidification of the outer shell of these MCs was achieved by lowering the pH further, here attributed the proton-induced interfibrillar interactions of OCNF networks.^30^ On the contrary, at higher pH (8.5), dye release increased to ∼6.4% (after 1 day) and ∼7.5% (after 7 days). This is tentatively attributed to the presence of Na^+^ ions (used to increase the pH), which screened the external negative charge on the microcapsules, causing significant aggregation, hence disrupting the OCNF/CCNF complex shell networks at the O/W interface.

The dye release of third MCs was also characterized under two distinct force fields, centrifugation and mechanical stirring. After centrifugation at 8000 rpm for 10 min at room temperature, the most stable third MCs showed 30 and 43% lower dye release than the primary PE after 1 and 7 days, respectively (Figure 5b). The dye release obtained via centrifugation was higher compared to the diffusion case. However, in the case of the third MCs, no remarkable increase in dye release was observed after 7 days compared to the first day, suggesting that after an initial release caused by the centrifugation, there was no further release due to diffusion through the cellulose shell. In addition, by lowering the pH, the third MCs showed that the release profile did not change after an additional 7 days of storage at room temperature (Figure 5d). Higher pH (8.5), on the other hand, led to further dye release after centrifugation and after extra 7 days of storage at room temperature, which further confirms the lack of stability of these MCs at higher pH.

**Figure 6:**
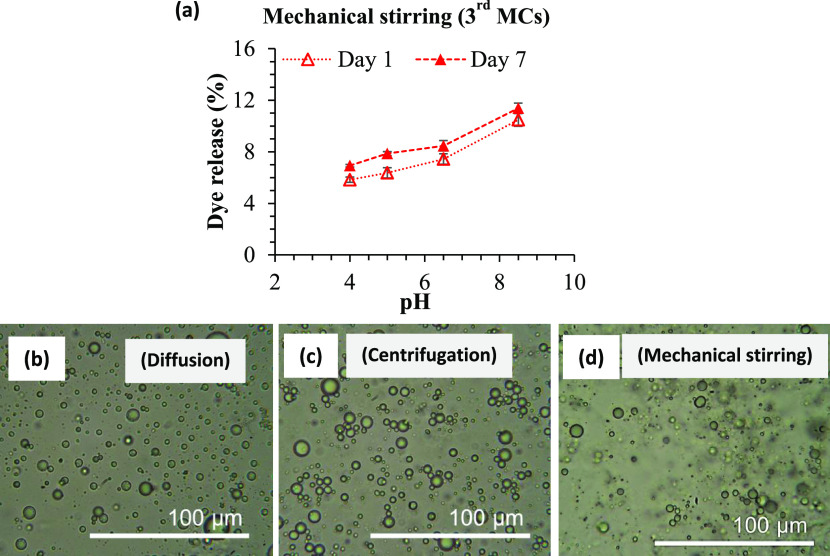
(a) Dye release from the third MCs obtained after stirring using an overhead mechanical stirrer (2000 rpm for 10 min) in various pH environments. (b–d) Optical micrographs show that the MCs are stable after diffusion, centrifugation, and mechanical stirring processes at control pH (∼6.5) after 7 days of storage at room temperature.

The robustness of the third MCs was further characterized by blending the MCs with fresh oil using an overhead stirrer at 2000 rpm for 10 min and then allowing the suspension to settle at room temperature. After 1 day of settling, the MCs and the free oil phases had not separated; as such, a short centrifugation (8000 rpm for 2 min) was used to separate the oil layer for the absorbance measurement (as shown in Figure S3). After 1 day, the dye release of these mechanically stirred third MCs was ∼5.8, 6.4, 7.4, and 10.5% at pH 4.0, 5.0, 6.5, and 8.5, respectively (Figure 6a). However, after 7 days of storage, the mechanically stirred MCs showed a higher release compared to day 1 for all pH environments investigated. The mechanical stirring process might have weakened some of the shell networks even in lower pH environments, causing the weaker capsules to coalesce and thus increasing the dye released over the storage period.

The third MCs showed a similar increasing trend of dye release with increasing pH environment for both the static (diffusion) and applied force field conditions (centrifugation and mechanical stirring) after 7 days of storage at room temperature. Although the mechanical stirring showed a higher release profile than the centrifugation and diffusion, the release percentage was still below 9% after 7 days at control pH (∼6.5). This suggests that, under these conditions, the third MCs are highly stable, as also evidenced from their optical micrographs (Figure 6b–d). This low dye release profile demonstrates that the MCs can withstand conditions used in a formulation, where the MC would be incorporated using mechanical stirring into commercial products. The full release of oil-soluble active ingredients (such as micronutrients or drugs) from these microcapsules could then be triggered via enzymatic degradation of the cellulose particles, e.g., if they are formulated for agricultural^32^ or pharmaceutical applications.^33^

### Microcapsules Prepared Using Membrane Emulsification

**Figure 7:**
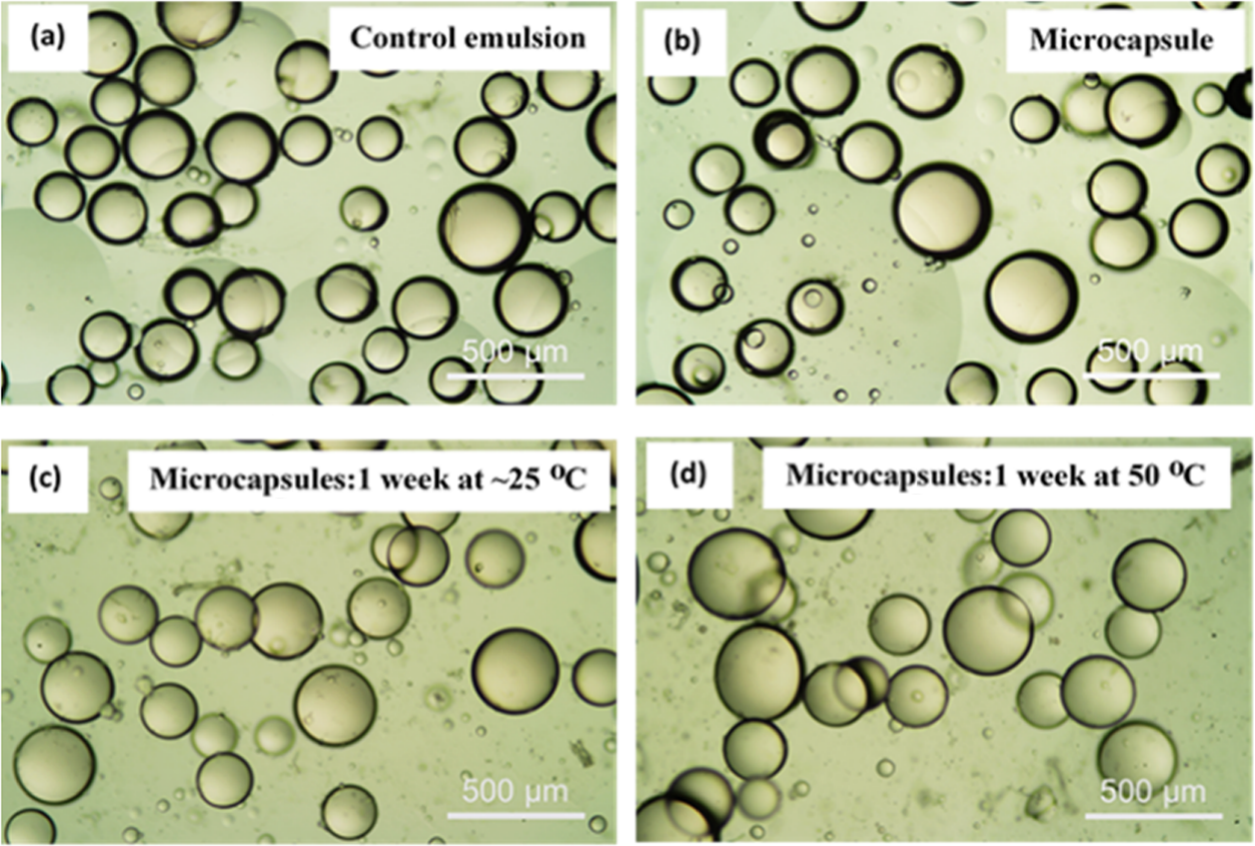
Micrographs of (a) control CCNF (0.1 wt %)-stabilized Pickering emulsion prepared upon adding sunflower oil (dispersed phase) to 0.1 wt % CCNF dispersion (continuous phase) via membrane emulsification; (b) microcapsules prepared by the addition of the OCNF dispersion to the CCNF (0.1 wt %)-stabilized primary PE, (c) microcapsules after storage for 7 days at room temperature, and (d) microcapsules after storage for 7 days at 50 °C.

The CCNF (0.1 wt %)-stabilized primary PE was also prepared using membrane emulsification to provide proof-of-concept for scaling up the microcapsule production. As NR was dissolved in sunflower oil and encapsulated by the CCNF/OCNF complex layer, the continuous phase for the membrane emulsification was the CCNF aqueous dispersion (0.1 wt %), and NR dissolved in sunflower oil was used as the dispersed phase, which was injected through a 30 μm pore membrane into the continuous phase. The oil droplets formed at the membrane permeate side were stabilized by the CCNF in the continuous phase, forming the primary PE. At the low rotation speed used (∼530 rpm), larger droplets (190 ± 35 μm, measured using ImageJ) were obtained, compared to the batch process (which was formed via high shear generated using an Ultra Turrax homogenizer), as expected (Figure 7a). After slowly adding the OCNF dispersion to the oppositely charged primary emulsion (maintaining the same OCNF/CCNF ratio as that in the third MCs prepared earlier), electrostatic interactions at the O/W interface led to the formation of microcapsules with an average diameter of 220 ± 48 μm (Figure 7b). No significant change in the size of the droplets was observed for the microcapsules even after storage at room temperature for 1 week (220 ± 60 μm) and at 50 °C (224 ± 65 μm) compared to the primary PE droplets (Figure 7c,d).

## Conclusions

This study utilized the electrostatic attraction of various concentrations of oppositely charged cellulose nanofibrils at the oil–water interface to form microcapsules. The most stable microcapsules were produced from the cationized cellulose nanofibrils (CCNF∼0.1 wt %)-stabilized Pickering emulsions after the addition of negatively charged oxidized cellulose nanofibril (OCNF ∼0.5 wt %) dispersions. Complex CCNF/OCNF networks formed at the oil–water interface via the charge inversion of the primary Pickering emulsion, which played a vital role in controlling the stability of the microcapsules against coalescence. The stability of these microcapsules was further enhanced by increasing the amount of OCNF to allow more interactions with the CCNF that anchored at the oil–water interface. NR dye release from the oil phase through the CCNF/OCNF complex shell networks via diffusion and centrifugation processes revealed that dye release decreased with increasing concentration of OCNF at the oil–water interface. In addition, mechanical stirring showed a higher dye release profile compared to the centrifugation and diffusion measurements; however, the release percentage was still below 9% (at pH 6.5) after 1 week of storage at room temperature. Dye release from these microcapsules was reduced by decreasing the pH of microcapsule suspensions due to proton-induced interfibrillar attractions between the OCNF present at the oil–water interface. At the higher pH (∼8.5), the stability of the microcapsules reduced. The microcapsules were also successfully produced via membrane emulsification as a proof-of-concept for potential scale-up, showing promise for the large-scale manufacturing of stable microcapsules produced using renewable cellulose and their use in the controlled release of oil-based ingredients.

## Supporting Information Available

Conductometric analysis of the degree of substitution of CCNF; calibration curve for absorbance of NR in sunflower oil; photographs showing the stages in the dye diffusion and release protocols used in this study; photographs of stable and unstable microcapsules; optical microscope images of microcapsule samples; images of the contact angle of water on glass slides coated with OCNF or CCNF; and summary of the OCNF and CCNF concentrations in the samples (PDF)
