# Supplementary material for: Stable Cellulose Nanofibril Microcapsules from Pickering Emulsion Templates
Source: Langmuir. 2022 Mar 9;38(11):3370–9. doi: 10.1021/acs.langmuir.1c03025 (PMC9007535; doi:10.1021/acs.langmuir.1c03025)
Supplement: Supplementary file 1 — la1c03025_si_001.pdf [file la1c03025_si_001.pdf]

SUPPORTING INFORMATION

Stable cellulose nanofibril microcapsules from Pickering emulsion templates

Hui Shi[ab], Kazi M. Zakir Hossain[ab], Davide Califano[ab], Ciaran Callaghan[bc], Ekanem E. Ekanem[c], Janet L. Scott[ab], Davide Mattia[c], and Karen J. Edler[ab*]

[a]Department of Chemistry, University of Bath, Claverton Down, Bath, BA2 7AY, UK

[b]Centre for Sustainable Chemical Technologies, University of Bath, Claverton Down, Bath, BA2 7AY, UK

[c]Department of Chemical Engineering, University of Bath, Claverton Down, Bath BA2 7AY, United Kingdom

[*]Corresponding author: K.Edler@bath.ac.uk

Conductometric analysis of the degree of substitution:

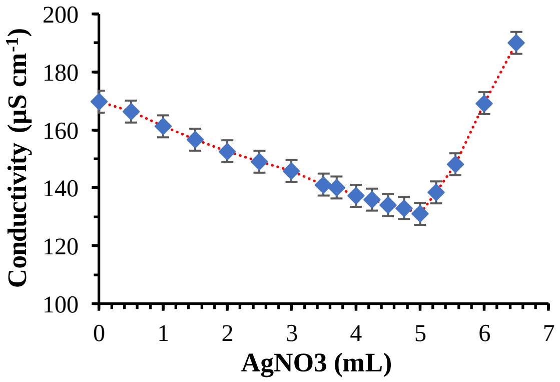

Figure S1. Conductivity curve for CCNF in DI water titrated with $AgNO_3$ (10 mM) aqueous solution.

The degree of substitution (DS %) of cationic cellulose was determined by conductometric

titration of chloride ions (trimethylammonium chloride groups) with $AgNO_3$ (aqueous) as described previously. [1, 2] The conductivity was monitored using a SevenMulti Mettler Toledo conductivity probe. The degree of substitution is calculated by using equation (S1):[1]

$$DS\ \% = \frac{162.15 * (c * V)}{m - (151.63 * c * V)} * 100 \dots\dots\dots\dots\dots\dots (S1)$$

Where c is the concentration of $AgNO_3$ solution (mol dm^{-3}), V is the volume of $AgNO_3$ solution (dm^3), and m is the weight of the dried cationized cellulose sample (g), 162.15 g mol^{-1} is the molecular weight (Mw) of the anhydroglucose unit (AGU), and 151.63 g mol^{-1} is the difference in Mw between the AGU and cationized AGU bearing trimethylammonium chloride groups. Triplicate samples were tested, and an average (with standard deviation) was reported.

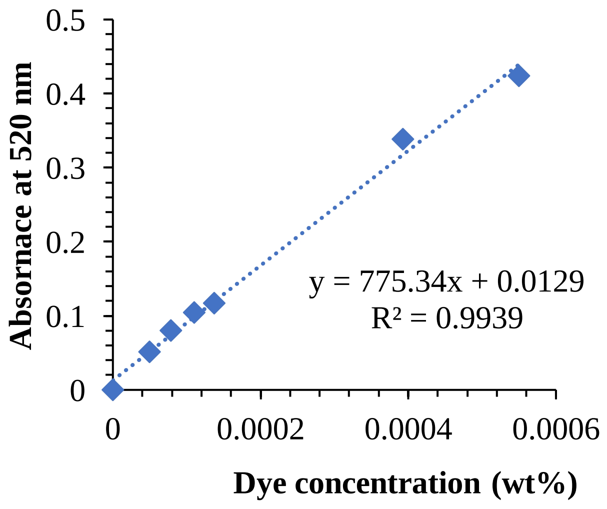

Figure S2. Calibration curve of Nile red (NR): absorbance at 520 nm as a function of NR concentration in sunflower oil.

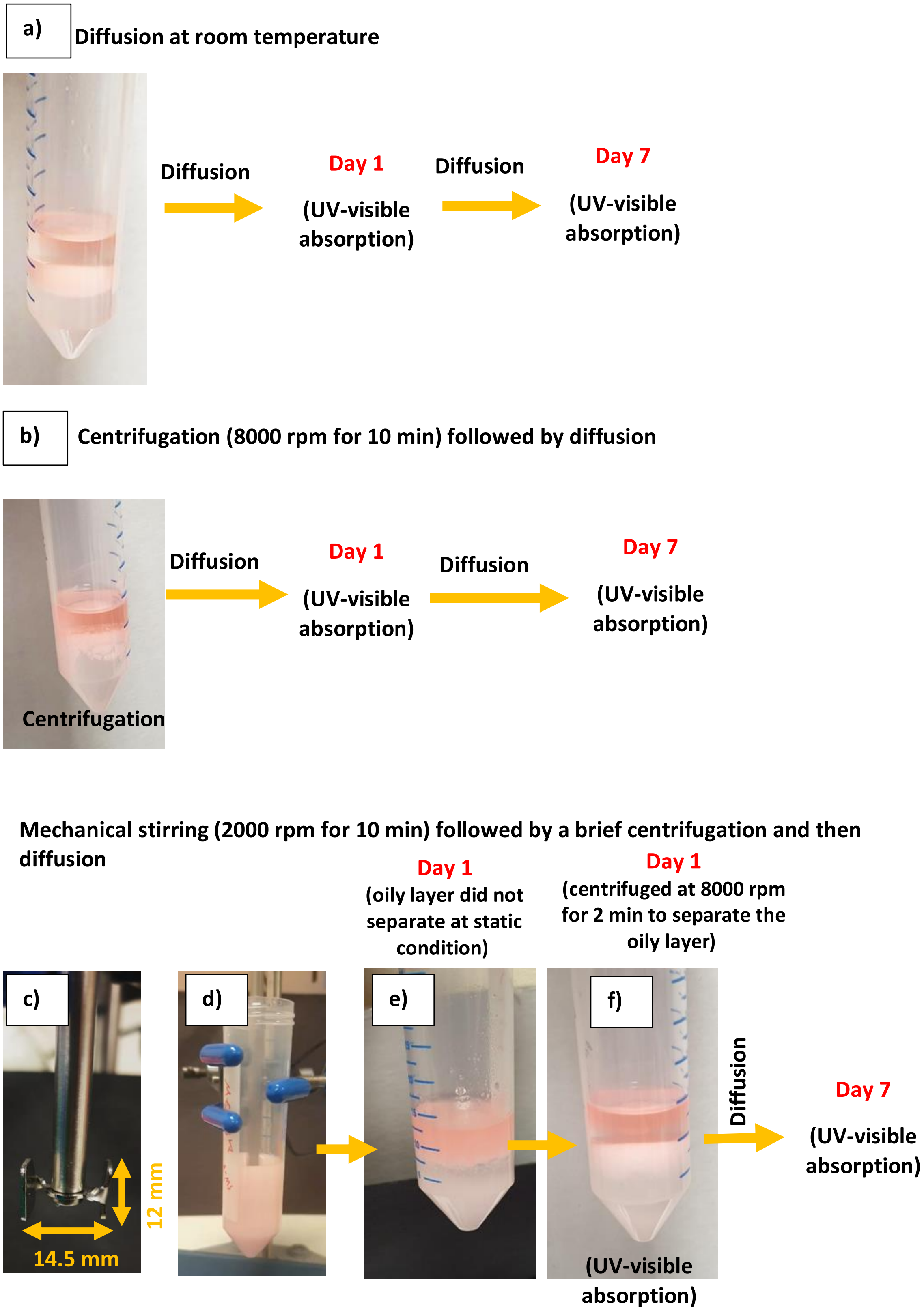

Figure S3: Photographs show the a) dye diffusion protocol used in this study, b) dye release to the free oil after centrifugation, c) dimension of the mechanical stirrer used for dye release study, d) mechanical stirring at 2000 rpm for 10 min, e) then after storing for 1 day at room temperature (did not show any separation of

clear oil phase) and f) after a brief centrifugation (8000 rpm for 2 min) allowed the separation of clear oil layer from the MCs (for UV-visible spectra analysis).

Contact angle of water

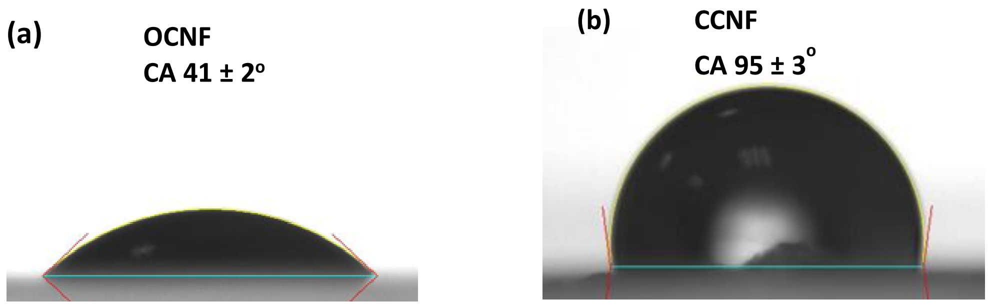

Figure S4. Contact angle (CA) of water on the glass slide coated with **(a)** OCNF and **(b)** CCNF.

The contact angle of water on the glass slide coated with OCNF and CCNF was measured using a drop tensiometer (OCA 20, Dataphysics Co., Germany) at ambient temperature. A 100 µl of the aqueous OCNF and CCNF dispersions (2 wt%) were placed on a glass slide and allowed to dry at 50 °C to form a thin film on the glass surface. A 10 µl of DI water drop was deposited via a microsyringe on the film surface, and the contact angle was measured immediately. The reported contact angle value was taken from an average of 5 measurements.

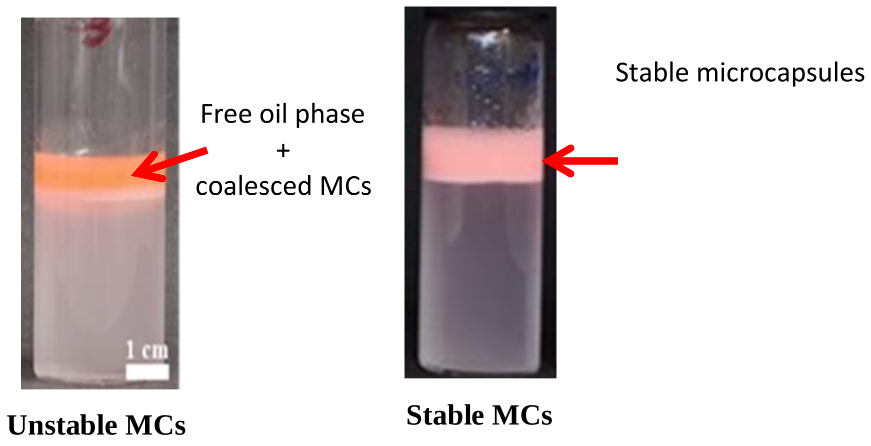

Figure S5. Photographs of the 'unstable' and 'stable' microcapsules: free oil phase was seen in the unstable MCs due to coalescence, while there was no free oily layer in the case of stable MCs. The cloudy aqueous phase (bottom layer) contained some OCNF/CCNF complexes that were not bound to the encapsulated oil droplets, which migrated to the upper layer due to the density difference.

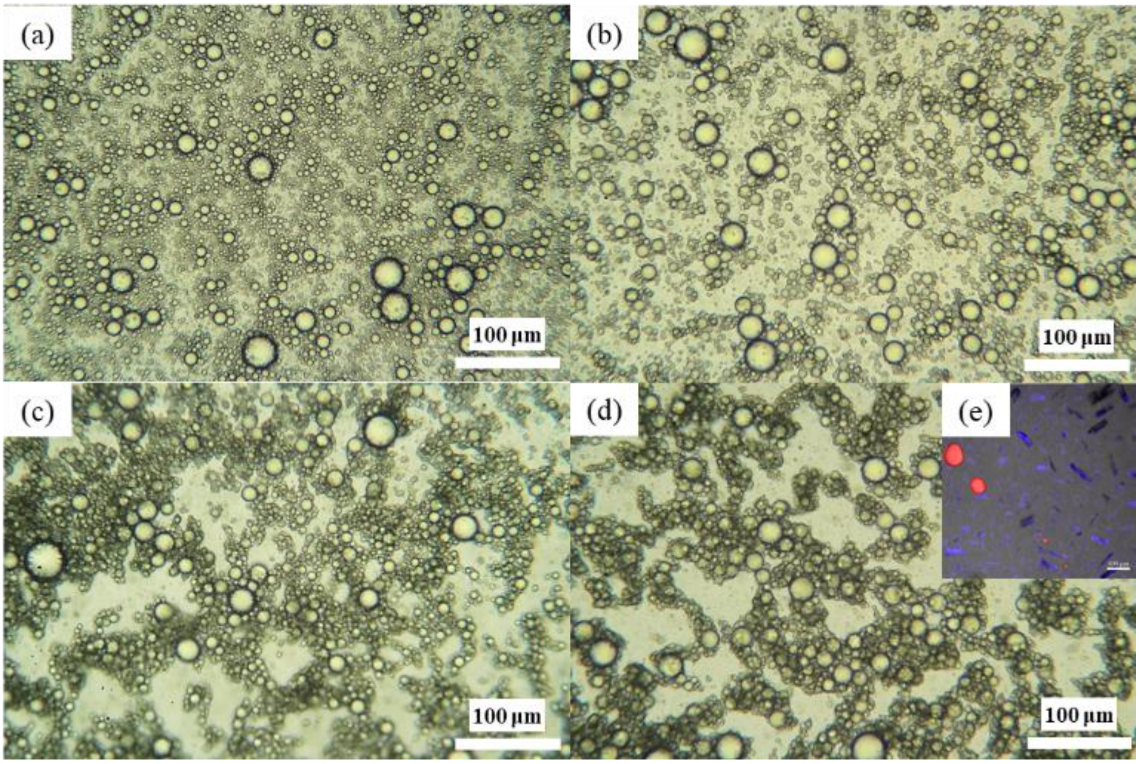

Figure S6. Optical microscope images of samples 1,2, and 3 generated by gradually replacing 1.5 mL of OCNF (0.5 wt.%)-stabilized emulsion with equal amount of 0.05 wt.% CCNF dispersion according to the method illustrated in Figure 3: (a) control OCNF-stabilized PE, (b) 1st microcapsule sample, (c) 2nd microcapsule sample, and (d) 3rd microcapsule sample. Images were taken just after preparation. (e) Confocal microscope image of 3rd microcapsule sample after standing at room temperature for 1 week (cellulose stained with calcofluor white stain showing blue and sunflower oil dyed with Nile red showing red).

Table S1. Summary of the OCNF and CCNF concentrations in PEs and various MC samples.

**CCNF stock (wt%)**	**OCNF stock (wt%)**	**Samples** (MCs were prepared according to the scheme presented in Figure 1 using CCNF and OCNF stock dispersions)	**Total particle concentration**		**Mass ratio= CCNF/OCNF**
			CCNF (wt%)	OCNF (wt%)	
0.05	0.05	PE	0.0450	-	-
		1st MCs	0.0383	0.0075	5.10
		2nd MCs	0.0325	0.0139	2.34
		3rd MCs	0.0276	0.0193	1.43
0.1	0.05	PE	0.0900	-	-
		1st MCs	0.0765	0.0075	10.20
		2nd MCs	0.0650	0.0139	4.68
		3rd MCs	0.0553	0.0193	2.87
0.1	0.2	PE	0.0900	-	-
		1st MCs	0.0765	0.0300	2.55
		2nd MCs	0.0650	0.0555	1.17
		3rd MCs	0.0553	0.0772	0.71
0.1	0.5	PE	0.0900	-	-
		1st MCs	0.0765	0.0750	1.02
		2nd MCs	0.0650	0.1388	0.47
		3rd MCs	0.0553	0.1929	0.29

REFERENCES

(1) Courtenay, J. C.; Johns, M. A.; Galembeck, F.; Deneke, C.; Lanzoni, E. M.; Costa, C. A.; Scott, J. L.; Sharma, R. I. Surface modified cellulose scaffolds for tissue engineering. *Cellulose* **2017**, *24* (1), 253-267. DOI: 10.1007/s10570-016-1111-y.
(2) Courtenay, J. C.; Ramalhete, S. M.; Skuze, W. J.; Soni, R.; Khimyak, Y. Z.; Edler, K. J.; Scott, J. L. Unravelling cationic cellulose nanofibril hydrogel structure: NMR spectroscopy and small angle neutron scattering analyses. *Soft Matter* **2018**, *14* (2), 255-263, 10.1039/C7SM02113E. DOI: 10.1039/C7SM02113E.
